# A clinician implementation protocol for BetterBrains: An online, person‐centred risk factor management program to prevent cognitive decline

**DOI:** 10.1111/ajag.70005

**Published:** 2025-02-24

**Authors:** Stephanie Pirotta, Anna Barker, Renata Morello, Emily Rosenich, Stephanie Perin, Samantha De Araugo, Yen Ying Lim, Darshini Ayton

**Affiliations:** ^1^ School of Public Health and Preventive Medicine Monash University Melbourne Victoria Australia; ^2^ Turner Institute for Brain and Mental Health, School of Psychological Sciences Monash University Clayton Victoria Australia

**Keywords:** Alzheimer's disease, cognitive dysfunction, dementia, risk reduction behaviour

## Abstract

**Objective:**

Modifiable risk factors, particularly those in midlife, can contribute to cognitive decline and dementia. Despite this, the ‘how’ of dementia risk reduction, including the application of interventional and care frameworks to deliver such a program is lacking. Our aim was to describe the ‘how’ in clinical delivery of a dementia risk reduction program called BetterBrains.

**Methods:**

BetterBrains is an online, person‐centred risk factor management program designed to prevent or delay cognitive decline in cognitively unimpaired community‐dwelling, middle‐aged adults with a family history of dementia. This protocol describes the delivery and implementation of BetterBrains using the Exploration, Preparation, Implementation, Sustainment (EPIS) framework.

**Results:**

Procedures for risk factor assessment and tailored management pathways using motivational interviewing, nationwide community linkage mapping and community referral pathways using digital delivery are outlined. Coach training and competency checks for program fidelity measures are also described.

**Conclusion:**

Complex, multi‐component programs require detailed implementation processes. Clinicians delivering BetterBrains may be better supported through standardised operating procedures, training and monitoring of competencies and implementation fidelity.

**Practice impact**

This BetterBrains clinical program will better train and support a dementia prevention workforce within community settings, helping reduce the risk and burden of dementia on public health‐care systems. The protocol provides a practical guide in how to best support dementia prevention in the community.

## 1 INTRODUCTION

Dementia, of which Alzheimer's disease (AD) is the most common form, is the seventh leading cause of disability and death worldwide,^1^ with an estimated annual global cost of US$2 trillion by 2023.^1^ By the year 2050, the incidence of dementia globally is projected to triple from 52 to 152 million,^2^ urging a timely and sustained investment in dementia care and prevention.^3^ Despite recent advances in AD therapeutics, there remains no accessible cure for dementia. However, even in the absence of a cure, the incidence of dementia in Western, high‐income countries is decreasing.^4^ This decrease has been attributed to successful public health campaigns targeting smoking, alcohol use and cardiovascular risk factors. As such, dementia risk reduction may be possible by targeting a range of modifiable dementia risk factors (MDRFs), including smoking, obesity, low mood, physical inactivity, low social contact, excessive alcohol consumption, hypertension, high cholesterol, diabetes, hearing impairment and depression.^5^ With this in mind, several randomised controlled trials (RCTs) have been conducted to determine whether improving MDRFs may lead to preserved cognitive function and reduced dementia risk, albeit with mixed results.

More recently, RCTs that target multidomain MDRFs, such as the Finnish Geriatric Intervention Study to Prevent Cognitive Impairment and Disability (FINGER),^6^ the French Multidomain Alzheimer Preventive Trial (MAPT),^7^ and Maintain Your Brain,^8^ have been conducted. These trials implemented a prespecified module trial design that targets each MDRF (e.g. poor nutrition, physical inactivity and low cognitive engagement) to be completed by all participants. As research continues to evolve, it is now well established that implementing a person‐centred health‐care approach is best to optimise program and clinical outcomes. Programs need to be adapted to a person's personal identity, health profile, environment, strengths, personal rights and future plans. Doing so has been associated with improved quality of life, personal and health‐care professional satisfaction, well‐being, reduced length of hospital stays, reduced health‐care costs and health‐care outcomes when compared to a predetermined health pathway across several conditions.^9^, ^10^ For example, program evaluation of RESPOND, a telephone‐based, person‐centred fall prevention program, showed high acceptability and increased participant engagement (83%), compared to standard usual fall prevention care in a sample of at‐risk older adults.^11^

The importance of dementia‐specific risk reduction programs and the availability of a trained public health workforce to target those at risk of dementia, reduce incidence rates and better manage dementia‐related health demands has been acknowledged in the Australian Federal Dementia Policy,^12^ the Royal Commission into Aged Care Quality and Safety,^13^ and the Human Rights Watch ‘Fading Away’ Report.^14^ The public health workforce should therefore also be trained in medical and non‐medical methods to best support these dementia‐related health demands, to meet the complex needs of people at risk of dementia in Australia.^15^ Recently, we described a novel person‐centred dementia risk factor management program that tailors the program to individuals' health needs and goals.^16^ The BetterBrains program utilises a person‐centred health coaching approach to best support positive dementia risk reduction‐oriented behaviour change. To do this, individual behaviour change determinants, such as health literacy, self‐efficacy, personal health goals, community resources, service and health‐care accessibility and personal financial position are considered.

Given the challenges associated with the implementation of a complex dementia risk reduction program, this protocol provides guidance on the practical application of BetterBrains in detail, complementing the overall description of the BetterBrains intervention protocol that has been previously published.^16^ The aims of this paper were therefore two‐fold. The first aim is to describe the role of the health coaches in complex dementia risk reduction programs and the skills required to fill current gaps in the provision of these programs. The second aim is to describe the planned practical application of the BetterBrains program (using the Exploration, Preparation, Implementation, Sustainment (EPIS) Framework) to prevent or delay cognitive decline in healthy community‐dwelling adults at risk of dementia.^17^ The EPIS Framework is one model commonly used to identify key factors and processes to facilitate the implementation of complex programs within the public sector and social and allied health service systems worldwide.^17^, ^18^

## 2 METHODS—BETTERBRAINS PROGRAM IMPLEMENTATION

A summary of the implementation of the BetterBrains program using the EPIS framework is outlined in Table 1. Focus was given to the implementation phase of this framework to help guide and support future public health workers and researchers into the practical application and set‐up of the BetterBrains program. This clinical implementation process was included in the ethics application for the RCT. Ethics approval was granted by Monash University Human Research Ethics Committee (Project ID: 25221).

**TABLE 1:** Summary of the BetterBrains program according to the EPIS framework.

	EPIS factor	Components in BetterBrains program	
Outer context	Funding	National Health and Medical Research (NHMRC) Boosting Dementia Research Initiative grant (GNT1171816)	
	Leadership	Chief investigators—neuropsychologists, neurologists, health innovation researchers, epidemiologists, sleep specialists, public health researchers, general practitioners, biostatisticians and computer scientists	
	Inter‐organisational environment and networks	Partnerships between ‘Removed for review’	
	Participant characteristics	Aged 40–70 years ≥1 risk factor for dementia Self‐reported willingness to make behaviour changes English speaking Access to the internet and a laptop or personal computer No neurological condition diagnosed	
	Participant advocacy	Connect with local community supports, for example general practitioners, specialists, allied health professionals, volunteering programs, sports clubs, Dementia Australia and online learning programs	
Innovation factors	BetterBrains program developers	Academics specialising in dementia risk reduction, sleep, psychology, nutrition, person‐centred models of care, implementation science and epidemiology Allied health clinicians: dietitians and psychologists Academic IT developers Research administration support	
	BetterBrains program characteristics	Risk factor assessment and management pathways, person‐centred care, digital delivery (Refer to Appendix S1)	
	BetterBrains fit	Improve poor sleep, low mood, low social and cognitive engagement and cardiovascular risk factors across the lifespan that are specific to dementia onset Person‐centred care Focus on reversing risk factors in midlife (40–70 years)	
Bridging factors	Community/academic partnerships	All free and fee‐paying services and organisations available in the community, for example general practitioner clinics, community centres, online programs, volunteering, sports, craft, language and conversation groups	
	Purveyors/intermediaries	Motivational interviewing: dietitians, psychologists Expertise of BetterBrains chief investigators: sleep therapy, neuropsychology, neurology, epidemiology, general practitioners, public health, health service development and improvement, program implementation and evaluation, cognitive assessment, biostatistics, data security and computing systems Allied health training of BetterBrains coaches: dietetics and psychology Program development and translation/implementation: public health scientists, BetterBrains coaches Evidence‐based recommendation synthesis for each domain according to narrative reviews or previously published systematic reviews.	
Inner context	Organisational characteristics	Culture: supportive, encouraging, flexible, person‐centred and evidence‐based Readiness for change: motivation to change questionnaire Leadership: 3 tiers (program developers, clinical managers and senior BetterBrains coach) Support for BetterBrains coaches: 4 × 60‐min case presentations and discussions per year; fortnightly 15‐min BetterBrains coach check‐ins Support for senior BetterBrains coaches: Monthly 30‐min meeting with clinical managers; fortnightly 30‐min meeting with research staff Support for participants: Instant messaging service to assigned BetterBrains coach or administration staff through website and/or smartphone app, online or phone sessions	

	Leadership	Chief investigator team—finalise program development, adverse event administration, financial contributions, guide overall BetterBrains team relating to program application, program duration Project management team—guide research administration and workload, oversee the application of the BetterBrains coaches and assist senior BetterBrains coach, finalise program design Senior coach—hire, train and manage the BetterBrains coaches	
	Quality and fidelity monitoring/support	Recording 20% of online sessions for auditing 1:1 meetings with BetterBrains coaches and BetterBrains senior coach every 2 months to discuss areas of strength and improvement regarding motivational interviewing and the BetterBrains protocol, guided by the RPAD Fortnightly data export to monitor: ⚬ Average participant time and administration time for each BetterBrains coach and overall team ⚬ Timing of coaching session and adherence to protocol ⚬ Number of GP letters sent ⚬ Number of baseline and follow‐up sessions completed to date and in the past fortnight ⚬ Number of goals developed and achieved overall and according to the targeted domain Engagement with monthly educational blog posts (‘BrainBlogs’) Frequency of weekly and monthly check‐ins completed	
	Organisational staffing processes	Motivational interviewing training Allied health or public health degree BetterBrains induction and use of BetterBrains coach platform	
Individual characteristics	Senior BetterBrains coach Allied health professional Clinical experience Trained in motivational interviewing Good communication skills Organised Leadership skills	BetterBrains coaches Allied health professionals Clinical experience Able to apply person‐centred care Positive attitude Open to learning Good communication skills Access to a computer and proficiency with online consultations

Abbreviations: EPIS, Exploration, Preparation, Implementation, Sustainment; NHMRC, National Health and Medical Research Council; RPAD, The Rochester Participatory Decision‐Making Scale.

The following are the *innovative factors* as part of the EPIS framework.

### 2.1 The BetterBrains program characteristics

**FIGURE 1:**
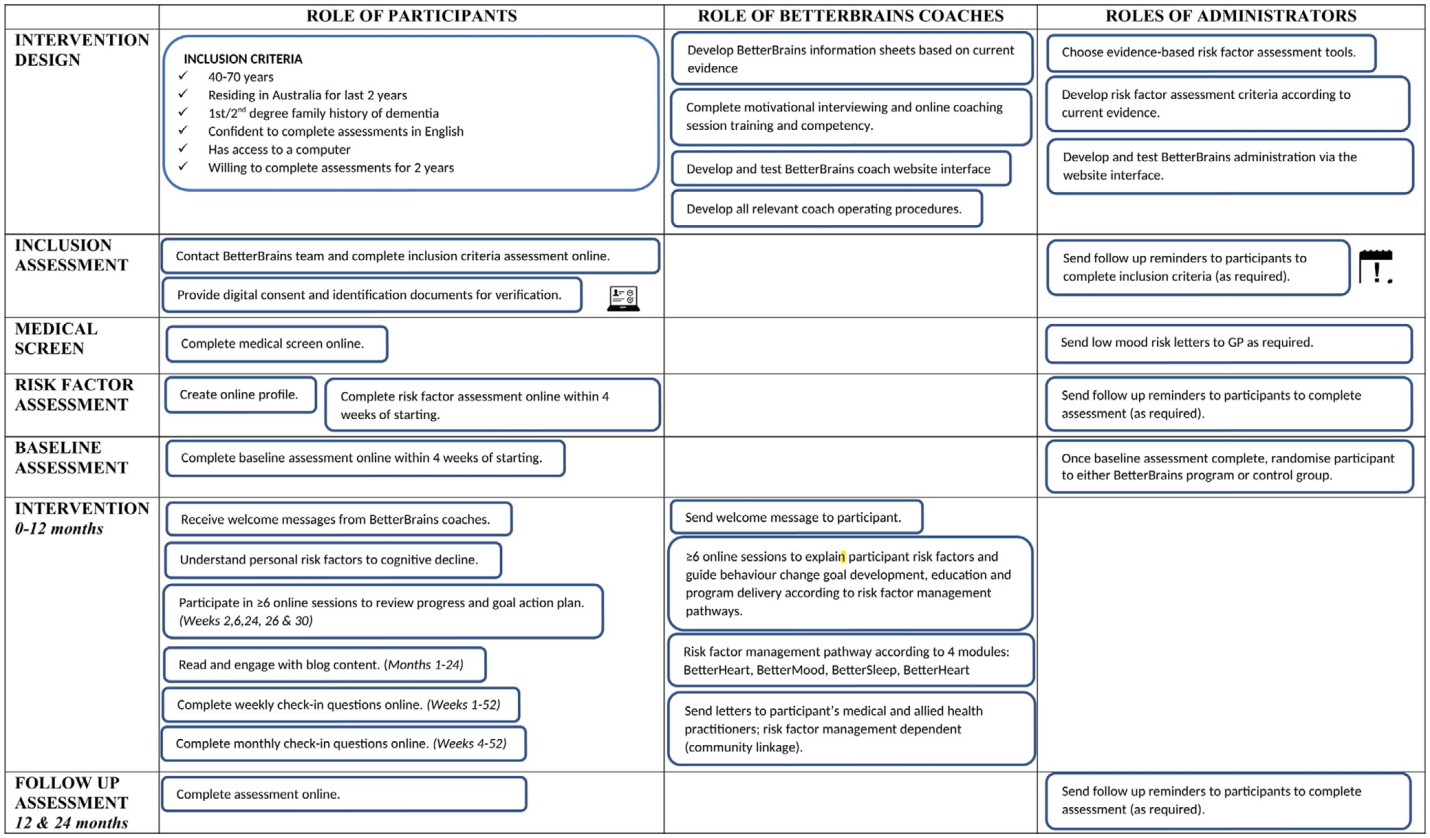
Overview of key activities within BetterBrains.

A detailed description of the BetterBrains intervention has been published previously.^16^ Briefly, BetterBrains is a 12‐month online, person‐centred risk factor management program that aims to prevent or delay cognitive decline in cognitively unimpaired community‐dwelling adults aged 40–70 years with a family history of dementia. Participants are required to undergo a comprehensive self‐report risk factor assessment across four broad risk domains (that also map to core program components, see Appendix S1), including cardiovascular health, cognitive and social engagement, mood symptomatology and sleep dysfunction. Participants are eligible to participate in the trial if they report at least one MDRF. Allied health professionals, called ‘BetterBrains coaches’, use motivational interviewing techniques to help participants develop positive health behaviours and set personal health goals targeting identified risk factors. A summary of the key activities included within the BetterBrains program is outlined in Figure 1.

#### 2.1.1 Risk factor assessment

To determine eligibility for the BetterBrains RCT, prospective participants are required to complete a comprehensive, self‐report risk factor assessment consisting of 11 questionnaires within 4 weeks (see Appendix S2 for the list of questionnaires included within the risk factor assessment). Participants must report at least one MDRF to be eligible for enrolment in the BetterBrains RCT. Risk categorisation, including a list of all risk factors assessed, corresponding risk factor domains and suggested goal development strategies, is outlined in Appendix S3. For further information, please refer to the original BetterBrains protocol paper.^16^

#### 2.1.2 Risk management pathways

A series of risk factor management pathways has been mapped onto each risk assessment outcome and has been broadly organised into the four core domains: BetterMood, BetterMind, BetterSleep and BetterHeart—targeting mood symptomatology, low social and cognitive engagement, sleep dysfunction and cardiovascular risk factors, respectively. For detail on each of these domains, refer to Appendix S3.

#### 2.1.3 Person‐centred care

BetterBrains coaches employ person‐centred care across the program. Person‐centred care involves changing behaviour through a collaborative, professional and equal partnership between the person and professional by developing tangible and realistic health‐care goals.^19^ This collaboration promotes sustainable behaviour change by increasing the person's skills and confidence to progress their health through regular assessment of progress and problems, goal setting and problem‐solving support.^20^ In line with this concept, the baseline session starts by providing the participant with an overview of the program. Next, BetterBrains coaches and the participant discuss the MDRF(s) identified within the participant's risk factor assessment. As part of this discussion, the participant is invited to share their reflections and opinions on these MDRF(s). Goals and strategies to modify identified MDRFs are developed collaboratively with the participant, considering personal interests, financial factors, health literacy/knowledge and the accessibility and availability of local resources and services, including travel capabilities. Participants are encouraged to connect with their support networks for additional motivation and encouragement, including family, friends, health professionals and community programs.

##### Motivational interviewing

BetterBrains coaches use motivational interviewing techniques across the four stages of motivational interviewing to implement person‐centred care (Table 2). Coach‐participant interactions are evaluated using the Rochester Participatory Decision‐Making Scale (RPAD).^21^ Motivational interviewing combines the Stages of Change Model and the Transtheoretical Model to facilitate health behaviour change.^22^, ^23^, ^24^, ^25^ The Transtheoretical Model utilises the Stage of Change construct and allows BetterBrains coaches to tailor the program to a participant's current stage of motivational readiness, including precontemplation (no intention to change behaviour within the next 6 months), contemplation (intention to change behaviour within the next 6 months but does not act on intention to change), preparation (intention to change behaviour within 30 days), action (changed from unhealthy to healthy behaviour within the past 6 months) and maintenance (maintenance of the behaviour change for more than 6 months).^25^, ^26^, ^27^ Progression between one stage and another is non‐linear and dependent on individual motivation, barriers and facilitators to change. The BetterBrains coach will modify communication and program methods according to a person's stage of change and personal circumstances during the 12‐month active phase.^25^, ^26^, ^28^

**TABLE 2:** Motivational interviewing stages and BetterBrains coach aims during the active intervention phase.

Motivational interviewing stage	Coach aim
Engage	Build rapport with the participant to establish a trusting and respectful therapeutic relationship and emphasise the importance of behaviour change to modify dementia risk factors Prioritise the participant and their perspectives so they feel supported, comfortable and safe
Focus	Determine how confident the participant is in regard to optimising behaviour to modify dementia risk factors Explore participant goals for change Determine participant goals and whether these target MDRFs to ensure there is a common purpose between the participant and BetterBrains coach Explore the participant's current knowledge and skills required to change behaviour and modify dementia risk factors Highlight current gaps in knowledge and skills needed to build participant momentum towards positive behaviour change to help modify dementia risk factors
Evoke	Encourage the participant to make positive behaviour changes by explaining the reasons for change and the positive outcomes they may have on cognitive decline
Plan	Collaboratively develop person‐centred and realistic goal(s) that consider the participants stage of change, knowledge, skills, motivation and circumstances to target MDRFs and prevent or delay cognitive decline

#### 2.1.4 Digital delivery

##### Website and smartphone application

Participants engage with the program and their BetterBrains coach through the BetterBrains website (Appendices S4 and S5) (www.betterbrains.org.au) and the free BetterBrains smartphone application (available on the Google Play and Apple App stores) (Appendix S6). Participants can observe their achieved and active goals, receive alerts for, and complete weekly and monthly check‐ins, receive assessment alerts, read monthly educational ‘BrainBlogs’ and send messages to their BetterBrains coach via both digital platforms.

##### Online coaching sessions

###### Initial coaching session

The initial session is an opportunity to meet and understand the motivators and intentions of the participant as well as clarify program expectations and inclusions over the next 12 months (Table 3). Questions are welcomed from the participant at any time. To help with this, the BetterBrains coach completes the initial assessment through a series of questions focusing on current lifestyle habits (volunteering history, nutrition, weight changes, recent lifestyle changes, recent falls and reasons for stress) and motivation to change. This all strengthens the coach‐participant bond that is essential for motivational interviewing and allows the BetterBrains coach to gain a better participant profile (Appendix S7).

**TABLE 3:** Description of BetterBrains coaching session content and considerations.

	Duration	Aim	Major components	Explanations and considerations
Initial coaching session	45 min	Outline program aim, inclusions and expectations and understand the participant health goals	Review participant profile (demographics, location, social history, medical history and medication use) as obtained in the baseline questionnaire prior to the session Overall welcome and BetterBrains program overview Discuss personalised MDRF(s) identified at risk factor assessment Initial coaching assessment (Appendix S7) Brief discussion of participant‐orientated behaviour change goals to target participant MDRFs Book in next session	Overall program aim Novelty to previous dementia prevention programs Major program components (BrainBlogs, weekly and monthly check‐ins) Coaching session frequency and duration as per protocol (minimum of six sessions) + per participant needs The BetterBrains smartphone application Use of the messaging system for communication How to schedule/reschedule future sessions using Calendly
Follow‐up coaching sessions	30 min	Develop a goal setting action plan that targets personalised MDRFs or reinforce previous goal setting	Identify lifestyle and medication changes since the last session Goal setting and/or goal review Information provided on MDRF(s) associated with cognitive decline Session summary Book in next session	Use motivational interviewing to facilitate: Positive health behaviour change Initiate linkages with community programs and support services Assess barriers to change Work through strategies to overcome these barriers Reinforce positive health behaviour to encourage self‐regulation and reduce cognitive decline At the beginning of each session prompt for information relating to changes since the last session: New medical diagnoses Medication or supplement regime changes Major life changes COVID‐19 diagnosis (if applicable, any associated implications, e.g. hospitalisation)

###### Follow‐up coaching sessions

During session 2, BetterBrains coaches recap the participant's identified MDRF(s) and prompt to understand which MDRF(s) the participant may wish to target (Table 3). The BetterBrains coach and participant then collaboratively develop tailored SMART (specific, measurable, achievable, realistic and timely) goals to target the chosen MDRF(s), with recommendations provided by the BetterBrains coach as needed, in accordance with the BetterBrains protocol (Appendix S3). As per motivational interviewing, participants are prompted to consider facilitators and barriers to change, with realistic suggestions discussed to help attain goals. Participants are also encouraged to self‐monitor when working towards their goal (e.g. using the BetterBrains smartphone app or paper‐based logging) to help increase overall motivation and track goal completion. All information provided during the session considers the participant's goal, available community resources, available financial resources and level of health literacy. Subsequent follow‐up coaching sessions incorporate these same components, with discussions centred around goal updates, barriers and enablers to changing behaviour, and, as needed, adapting or developing new SMART goals in response to a participant's MDRF(s).

##### Adherence strategies—participant check‐ins

Participants are presented with weekly and monthly check‐in questions. Weekly check‐ins assess self‐reported goal adherence and barriers to change and provide participants the opportunity to contact their BetterBrains coach (Appendix S8). Monthly check‐ins assess self‐reported changes in physical activity, cognitively stimulating leisure activities, sleep and diet quality, mental well‐being, smoking frequency, alcohol consumption, health consultations and the impact of the COVID‐19 pandemic on goal progress and behaviour change (Appendix S9). All check‐ins are completed through the BetterBrains smartphone application (Appendix S6). Participants are also sent regular email and SMS notification reminders to encourage weekly and monthly check‐in completion and trial engagement.

##### Information provision

When required, BetterBrains coaches will send additional information via the BetterBrains messaging system to participants to guide behaviour change or increase participant knowledge. Additional information may include links to websites with evidence‐based information or digital fact sheets. The digital fact sheet content is developed by researchers and clinicians considered experts in their fields working as part of the BetterBrains team or predeveloped from evidence‐based organisations (e.g. Dementia Australia, Heart Foundation, Sleep Health Foundation).

###### BrainBlogs

Monthly educational blogposts, called ‘BrainBlogs’, are sent to all participants through the smartphone application. BrainBlogs are written and produced by the BetterBrains research team, intending to facilitate trial engagement and educate participants on the latest developments in dementia and ageing research using easy‐to‐understand terminology and a lay writing style. To minimise interference with the program, BrainBlogs do not provide information about specific lifestyle changes or dementia risk reduction strategies that may reduce modifiable dementia risk. Topic examples include ‘What is memory anyway?’, ‘Biology of sleep and Alzheimer's Disease’ and ‘Neuroinflammation and dementia’.

### 2.2 Timing, delivery and mode

A minimum of six follow‐up coaching sessions are completed at 2, 6, 24, 26 and 30 weeks (±14 days) following the baseline session date, with additional sessions available upon participant request. Initial sessions comprise 45 min of participant‐facing time and 30 min of administration time (completing participant forms on the BetterBrains clinician interface, additional notes, sending summary messages to the participants and any referral letter(s), booking administration, as required). Follow‐up sessions are 30 min, with 15 min of administration time. Participants are allocated one BetterBrains coach over the 12‐month program unless otherwise indicated (e.g. BetterBrains coach on leave, participant requests a coach change or there is a conflict of interest). Sessions are conducted through Zoom or on the phone to promote easy nationwide access for participants.

The following are the *bridging factors* as part of the EPIS framework.

### 2.3 Community partnerships

#### 2.3.1 Community linkage

Connection to community services improves engagement in health‐based interventions and promotes positive lifestyle change, personal well‐being and health‐care satisfaction.^29^ Referral to community programs (online and/or in‐person) is encouraged during the BetterBrains program to help meet developed SMART goal(s) and support continuity of care once the intervention concludes. Community programs may include walking groups, art classes, mindfulness workshops, education classes and sports groups. The extent to which community linkage is successfully integrated into the BetterBrains program depends on the availability of local services/resources, participant motivation, financial factors and identified MDRFs.

Before starting the BetterBrains program, participants provide informed consent for their nominated health professional (e.g. GP, allied health or specialist practitioner) to be contacted with any relevant information. BetterBrains coaches send letters to the participant's nominated health professional when a behaviour change goal or strategy requires the input/management of professional health services (e.g. letter to GP recommending referral to sleep specialist due to sleep problems and previously diagnosed sleep disorder; letter to GP recommending exercise physiologist sessions under health‐care plan due to high cholesterol diagnosis) (Appendix S10). Letters to health professionals provide background information about the BetterBrains trial, a summary of participant MDRF(s) and general recommendations in response to presenting MDRF(s) and behaviour change goals and strategies.

The following are the *inner context factors* as part of the EPIS framework.

### 2.4 Quality and fidelity

#### 2.4.1 Participant engagement

Attrition and loss of engagement in behaviour change trials can be high.^30^, ^31^ To minimise attrition, participants receive reminder messages and calls to book and attend scheduled coaching sessions. Reminder messages are sent three and 7 days following the initial message sent to the participant to book in their session. Following no response, a final reminder call by the BetterBrains coach is completed 10 days following the date that the initial invite was sent. The participant is considered ‘disengaged’ if no response is received within 24 h of the final reminder call. Disengaged participants may return to the BetterBrains program at any time throughout the 12 months. The 12‐month timeline begins on the date of the initial baseline call.

#### 2.4.2 BetterBrains coaches

##### BetterBrains coach characteristics

BetterBrains coaches are allied health professionals clinically trained across a variety of disciplines (e.g. dietitians, psychologists, physiotherapists, exercise physiologists and nurses) or are public health workers (e.g. research fellow and public health researcher) with particular experience in motivational interviewing and person‐centred care. Personal characteristics sought when hiring for these positions included optimism, willingness to learn, well‐developed written and verbal communications skills, information technology familiarity, and knowledge of community health services and referral pathways. The core responsibilities of the BetterBrains coaches are described in Table 4.

**TABLE 4:** BetterBrains coach roles and responsibilities.

Responsibilities	Key activities
All BetterBrains coaches	
Delivery of BetterBrains program to participants	Review baseline assessments and highlight/discuss key MDRF(s) with participants Complete motivational interviewing to explore participant knowledge, beliefs, self‐efficacy, motivation to change and self‐directed strategies to reach personalised goals relating to the prevention of cognitive decline Facilitate the development of person‐centred SMART goal(s) and behaviour change strategies to modify MDRF(s) as part of the participant coaching process Schedule and complete follow‐up coaching sessions in support of participants achieving their SMART goal(s) Communicate with participants outside of scheduled coaching calls, as required for the BetterBrains program. Coaches should respond within 3 days of receiving participant messages Assist participants to navigate the health‐care system to address their MDRF(s) and achieve the planned SMART goal(s) Refer participants to community‐based services that complement the identified SMART goal(s) and risk factor management pathway Communicate with participant GPs and other health‐care professionals regarding identified MDRF(s) and action plans to achieve developed SMART goal(s)
Data collection	Initial assessment during the first session, via the BetterBrains coach interface Maintain participant confidentiality Participate in BetterBrains coach compliance checks for program delivery Complete participant forms for each coaching session, via the BetterBrains coach interface
Communication	Communicate to the Senior BetterBrains coach any program or participant issues Collaborate with other BetterBrains coaches as required (e.g. available resources, querying difficult participant presentations, crisis management support) Communicate with the administration team about participants wanting to discontinue the program or an adverse event Attend fortnightly BetterBrains coach meetings to discuss queries or program updates
Senior BetterBrains coach	
Intervention documentation	Contribute to the development of the BetterBrains coach training manuals and standardised operating procedures manual Train coaches in program delivery: motivational interviewing, clinical assessment, program practices and domains Assist with the monitoring of the BetterBrains data collection and participant engagement to complete assessments Attend and coordinate meetings involving external and internal stakeholders (clinical and research project staff, team, steering committee and investigator meetings), including preparation of agendas, papers, minute and correspondence throughout the program timeline Weekly export, save and securely store data relating to coach KPIs and present these at the coach meetings for regular review Check the ‘participant complaints’ Excel document monthly to review any areas of coach improvement
Leadership	Address clinical queries or questions that may arise during the planning and delivery of the BetterBrains program Coordinate and facilitate correspondence and meetings across all BetterBrains coaches to review issues relating to clinical practice and processes as they arise Support competency reviews of program practice across BetterBrains coaches Coordinate BetterBrains coach working hours and timetable, including leave management to ensure sufficient personnel coverage Communicate project issues to the principal investigators promptly Organise and lead fortnightly BetterBrains coach meetings
KPI completion	Respond to participant messages on behalf of other coaches, as needed Coordinate coach caseloads, ensuring scheduled calls are completed as scheduled Meet with BetterBrains coaches every 2 months to help BetterBrains coaches best meet motivational interviewing techniques Review the frequency and timing of participant coaching sessions according to program protocol

##### BetterBrains coach roles and responsibilities

BetterBrains coaches are primarily responsible for the delivery of the BetterBrains program (Table 4). A senior BetterBrains coach is responsible for coach recruitment, training, and coordination to ensure optimal program fidelity and data collection (Table 4). As BetterBrains is not a clinical service, BetterBrains coaches do not provide or guide medical management. Participants are referred to their nominated GP for medical management. In the presence of any psychological risk reported by the participant (e.g. suicidal ideation), coaches are trained to follow the ‘Managing Psychological Risk’ protocol (Appendix S11). The protocol aims to determine the overall level of suicidal risk and to determine the appropriate cause of action (e.g. calling the Crisis Assessment and Treatment Team or police in high to moderate situations or providing appropriate contact numbers for additional support services when presenting with low risk). In all cases, a follow‐up letter to the participant's GP is sent.

##### BetterBrains coach training

To enhance program fidelity and ensure consistency between coaches for the delivery of the BetterBrains program, all BetterBrains coaches complete a series of training sessions (in‐person and online) before the program commences and whilst working as part of the BetterBrains team (Appendix S12). Training covers the overall BetterBrains program design and aims, risk factor management pathways, BetterBrains domains, research considerations (e.g. fidelity), community linkage and suggested resources, BetterBrains coach interface and critical care protocols (participant crisis management, reporting of serious adverse and adverse events, and external BetterBrains coach supports). Training consists of an initial 3‐day intensive course followed by a 1‐day case study training and a half day of shadowing the BetterBrains senior coach completing coaching sessions (Appendix S13). Coach performance is monitored through competency assessments every 2 months to maintain a high level of program fidelity and inform the BetterBrains process evaluation.^32^ In the instance that additional training is necessary for any BetterBrains coach, one‐on‐one assistance through practical case studies with the senior BetterBrains coach is recommended. BetterBrains coaches meet weekly for the first 1–2 months and then fortnightly to discuss difficult cases, administrative matters, coach performances, program issues and strategies to overcome challenges as part of the BetterBrains program.

#### 2.4.3 BetterBrains coach competencies

Ensuring coaches are competent in completing their roles and are following specified protocols is important for optimal program evaluation and fidelity. BetterBrains coaches are assessed and monitored before and during the trial to ensure protocol adherence either online or in‐person. BetterBrains coach competencies are completed before commencement and are reviewed every 2 months (Appendices S14–S17).

### 2.5 Program strengths and limitations

This program has several strengths. Firstly, BetterBrains provides an easy‐to‐access opportunity for healthy individuals at risk of dementia to receive personalised, evidence‐based information and health coaching using phone or internet. This makes the program much more accessible across Australia, and possibly worldwide, helping reach remote and vulnerable population groups. It is important to note, however, that BetterBrains was designed for the general Australian population, and further tailoring is needed for other population groups, such as Culturally and Linguistically Diverse populations or Indigenous groups. Further development and evaluations informed by people of lived experience from these populations are needed to best tailor and meet individual group needs, considering culture, language and modes of delivery. Secondly, through taking part in BetterBrains, participants may be informed that they need to access specialist medical and/or allied health services to meet their needs. These services may require an additional financial cost at the expense of the participant.

For those wanting to implement BetterBrains, a few considerations are needed. Firstly, the use of a platform to assess participant risk factors, monitor progress, record participant notes and store personal information in a secure and legal manner is needed and is likely to come at a cost. A messaging system to connect with participants outside of coaching sessions is also highly recommended, and it is best to have this integrated into the same platform to maintain privacy. The use of phone and online telehealth platforms to connect and communicate with participants during the session is also needed and must be factored into the budget.

## 3 CONCLUSION

This paper details the core components of implementing the BetterBrains program from the perspective of BetterBrains coaches, including required characteristics and skills, preprogram delivery training, and ongoing competency checks to enhance program fidelity. It highlights the consideration and process that should be applied to ensure that the implementation and delivery of complex health interventions are as effective as they can be and that program outcomes and impacts are therefore optimised.

Should the BetterBrains trial be successful, the detailing of these components outlined in this paper will help future researchers and clinicians operationalise the BetterBrains program globally, using digital delivery services to prevent cognitive decline. In doing so, a public health dementia prevention workforce may be trained, helping to reduce the burden of dementia worldwide.

## FUNDING INFORMATION

The BetterBrains trial is funded by a National Health and Medical Research (NHMRC) Boosting Dementia Research Initiative grant (GNT1171816). Yen Ying Lim is supported by an NHMRC Career Development Fellowship (GNT1162645) and an NHMRC Investigator Grant (GNT2009550). Darshini Ayton is supported by an NHMRC Investigator Grant (GNT1195357). Emily Rosenich is supported by an Alzheimer's Association Research Fellowship (23AARF‐1025519).

## CONFLICT OF INTEREST STATEMENT

No conflicts of interest declared.

## TRIAL REGISTRATION

Registered in the Australian New Zealand Clinical Trials Registry (www.anzctr.org.au; ACTRN ‘removed for review’) on 20 April 2021.

## TRIAL STATUS

The trial is currently live and will continue until August 2025.

## Supporting information


Appendices S1–S17

## Data Availability

Data sharing is not applicable to this article as no new data were created or analysed in this study.
