# Supplementary material for: A clinician implementation protocol for BetterBrains: An online, person‐centred risk factor management program to prevent cognitive decline
Source: Australas J Ageing. 2025 Feb 24;44(1):e70005. doi: 10.1111/ajag.70005 (PMC11849804; doi:10.1111/ajag.70005)
Supplement: Supplementary file 1 — Appendices S1–S17 [file AJAG-44-0-s001.docx]

**Appendix S1:** BetterBrains core program components

1. **Risk factor assessment and management pathways**
2. Cardiovascular health ->BetterHeart
3. Low social and/or cognitive engagement ->BetterMind
4. Mood symptomatology -> BetterMood
5. Sleep dysfunction ->BetterSleep
6. **Person-centred care**
   - Motivational interviewing
   - Ongoing engagement (e.g. weekly and monthly check-ins)
   - Community linkage (e.g. community programs or referral letters to general practitioner, specialists)
7. **Digital delivery**
   - BetterBrains website and mobile application
   - Online sessions for goal development with BetterBrains coach (at least 6 across the 12-months through Zoom)
   - Information provision (monthly blogs, fact sheets as required)

**Appendix S2:** Questionnaires used for the BetterBrains risk factor assessment

| Mood symptomatology | - Depression, Anxiety and Stress Scale (DASS) (21-item) - Centre for Epidemiological Studies, Depression Scale (CES-D) - Hospital Anxiety and Depression Scale (HADS) |
| --- | --- |
| Low social and/or cognitive engagement | - Relationships Questionnaire - Educational and Occupational History |
| Sleep dysfunction | - Epworth Sleepiness Scale (ESS) - Insomnia Severity Index (ISI) - Berlin Sleep Apnoea Questionnaire (BQ) - Advanced Sleep Phase Questionnaire |
| Cardiovascular health | - International Physical Activity Questionnaire (IPAQ) - Medical and health history (smoking, alcohol intake) |

**Appendix S3:** BetterBrains risk factor assessment and management pathway

|  | **Risk Factor** | **Risk Assessment** | **Intervention options** | **Goal** |  |
| --- | --- | --- | --- | --- | --- |
| Cardiovascular Health | Atrial fibrillation | Self-reported (past/present) | - Medication review with GP reduce dietary salt intake - Increase adherence to MIND diet - Increase activity | - Medication adherence - Health & anthropometric targets | BetterHearts |
|  | Hypertension | BP>140/90 |  |  |  |
|  | Diabetes | Self-reported |  |  |  |
|  | High cholesterol | Self-reported |  |  |  |
|  | Obesity | BMI ≥ 25kg/m^2^ | - Increase steps/day - Engage in community gyms - Reduce sitting time | - Build to 6000-10,000 steps - Diet modification |  |
|  | Physical inactivity | <150 min/week of moderate intensity |  |  |  |
|  | Smoking | Current smoker | - Nicotine patches - GP smoking cessation plan | - Reduce/cease smoking |  |
|  | Excessive alcohol | >2 standard drinks/day | - Alcoholics Anonymous - GP cessation plan | - Target alcohol-free days/week |  |
| Social/cognitive engagement | Low educational attainment | Self-reported <12 years of education | - Participate in cognitively stimulating activities e.g. U3A, TAFE, Language classes | - Learn new or improve an existing skill - Engage in continuing education | BetterMinds |
|  | Monolingualism | Score 0 on language questionnaire |  |  |  |
|  | Social isolation | Social network score <10 | - Volunteer - Socialize with network | - Contact with social network >1/week |  |
| Mood | Elevated anxiety and depression symptoms | DASS-21: Depression >7; Anxiety >6; Stress >10  HADS-D/HADS-A: >8  CEDS: >16 | - Medical Management Facilitation/ Attending Psychology services (i.e. referral and engagement in health services through GP) - Behavioural Activation/Lifestyle (i.e. Physical activity & mindfulness | - Reduce depressive/anxiety symptoms - Learn self-management skills/strategies for symptoms - Improve overall wellbeing | BetterMood |
|  | Elevated stress symptoms |  |  |  |  |
| Sleep | Obstructive Sleep Apnoea | ESS (>8) on 2 out of 3 categories | - Intervention guided by formal sleep assessment | - Improved score on ESS | BetterSleep |
|  | Insomnia | ISI (>8) | - CBT-I -A Mindful Way. - Adherence/reduction guided by GP | - Improved score on ISI - Reduced medication use |  |
|  | Medication use | Weekly use >2 |  |  |  |
|  | Advanced Sleep Phase | Risk score on Advanced Sleep Phase Questionnaire | - Light therapy; Sleep monitoring (diary) | - Improved score on Advanced Sleep Phase Questionnaire |  |
|  | Daytime sleepiness | ESS >11 |  |  |  |

**Appendix S4:** BetterBrains coach website homepage

**
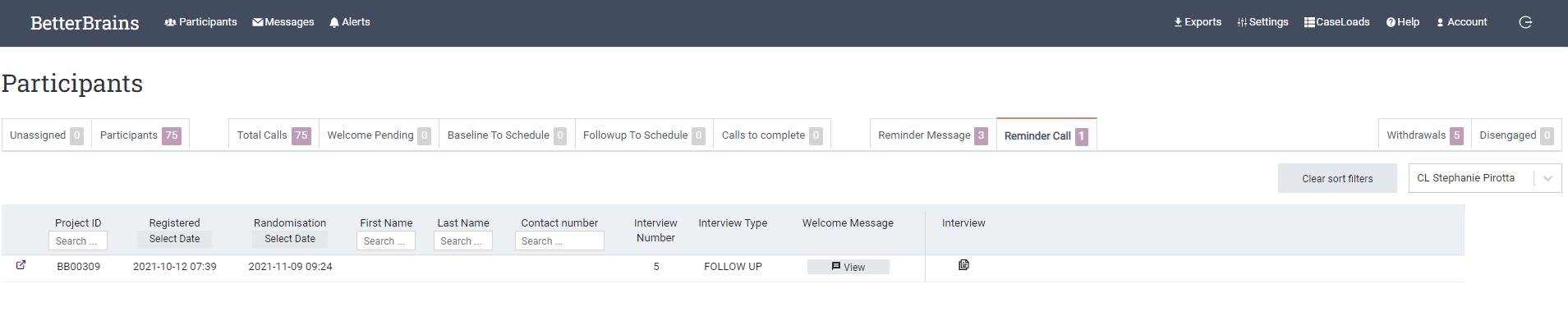
**

**Appendix S5:** Follow up call BetterBrains coach interface

**
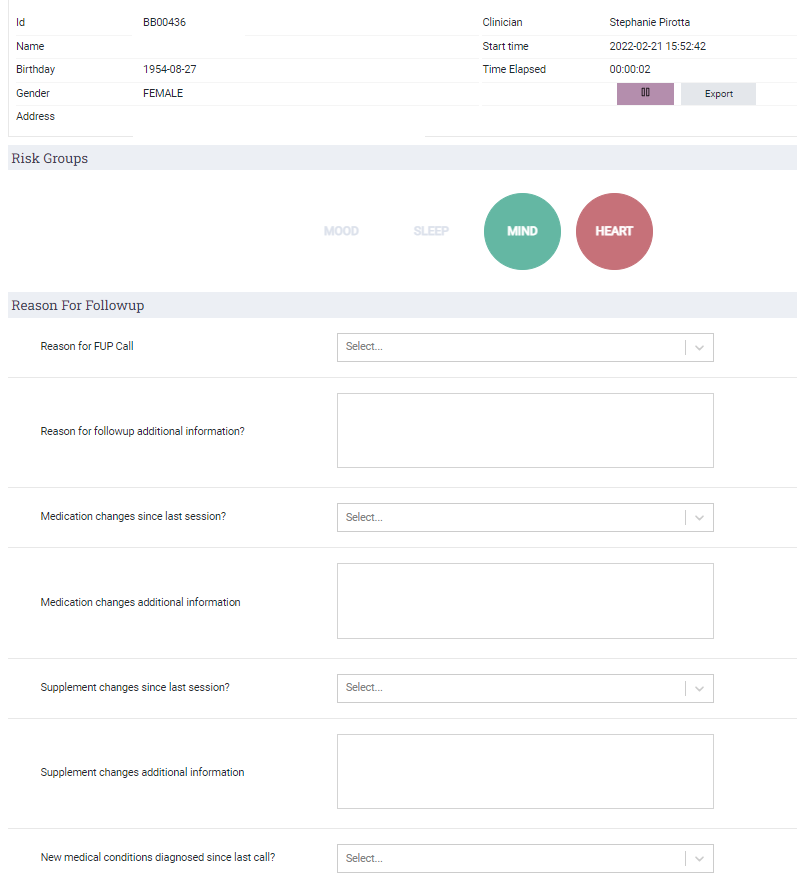
**

**Appendix S6:** BetterBrains smartphone app

**
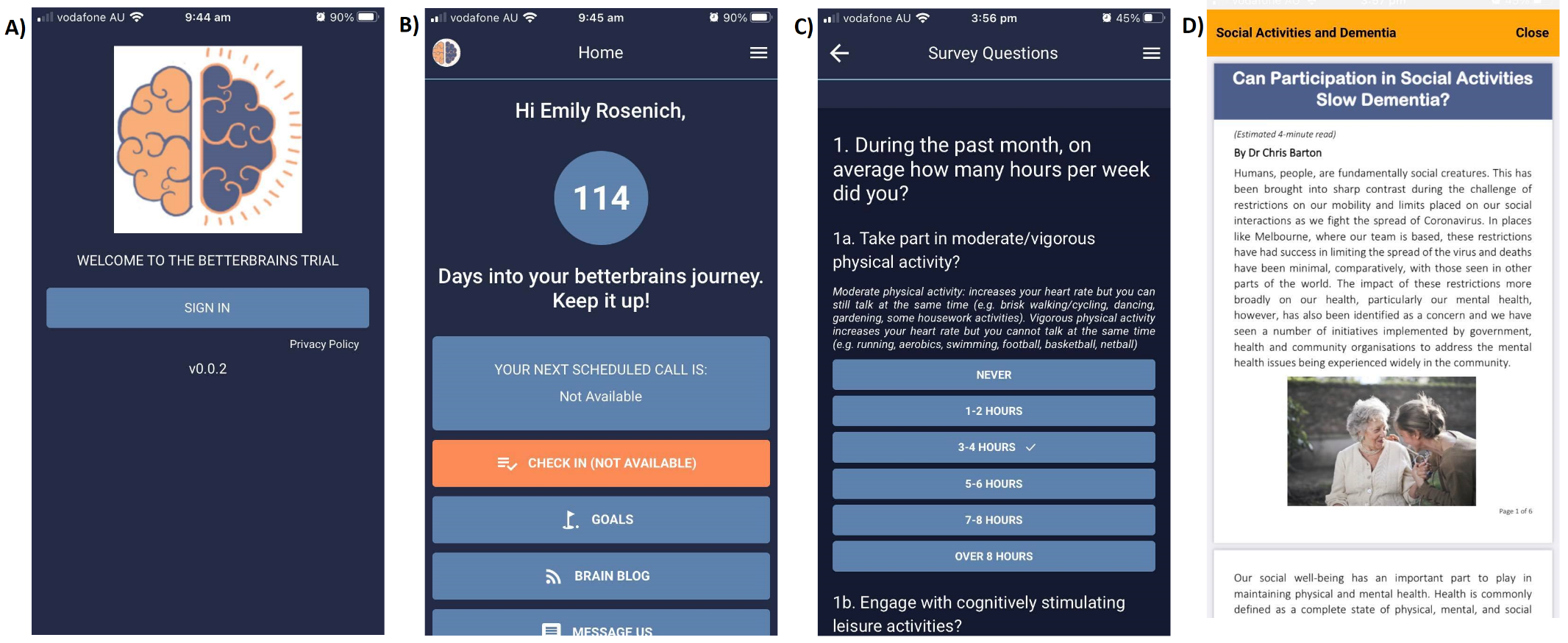
**

**Appendix S7:** Clinical assessment according to each BetterBrains module

| **Question** | **Assessed in baseline questionnaires** | **Assessed during initial session by the BetterBrains coach** |
| --- | --- | --- |
| ***Social history*** |  |  |
| What is your current relationship status? | ✓ |  |
| What is your current employment status? | ✓ |  |
| What was/is your current occupation? | ✓ |  |
| What are your living arrangements? | ✓ |  |
| Do you have any dependents? | ✓ |  |
| What is your ethnicity? (add additional information if required) | ✓ |  |
| What is your highest level of education? | ✓ |  |
| Do you volunteer in the community? |  | ✓ |
| Do you provide care to others who are not your dependents? |  | ✓ |
| ***Lifestyle*** |  |  |
| What are your current physical activity habits? | ✓ |  |
| How many hours on average are you sedentary during the day? | ✓ |  |
| Do you drink alcohol?  If yes, how many standard drinks on average per week? | ✓ |  |
| Do you smoke? | ✓ |  |
| Why did you start this Better Brains program? |  | ✓ |
| What are the top 3 health priorities you want to focus on at this point in your life? |  | ✓ |
| Have you recently made any changes to your lifestyle? |  | ✓ |
| What factors contribute to stress or anxiety in your life? |  | ✓ |
| Do you follow a particular diet? |  | ✓ |
| On average, how much water do you drink per day? |  | ✓ |
| On average, how many cups of coffee do you drink per day? |  | ✓ |
| On average, how many energy drinks do you have per week? |  | ✓ |
| ***Anthropometry*** |  |  |
| What is your weight? | ✓ |  |
| What is your height? | ✓ |  |
| BMI |  | ✓ |
| How has your weight changed over the last few years? |  | ✓ |
| What do you think has contributed to your change in weight? |  | ✓ |
| ***Clinical*** |  |  |
| Are there any medical conditions that run your family? | ✓ |  |
| Are you currently taking any medications? | ✓ |  |
| Are you currently taking any supplements? |  | ✓ |
| Do you currently have any injuries? |  | ✓ |
| Have you had any falls/slips or trips in the last 12 months? |  | ✓ |
| ***Diagnosis*** |  |  |
| Are you currently suffering from any medical diagnosis other than these? | ✓ |  |
| Is chronic pain a problem for you? | ✓ |  |
| Allergies | ✓ |  |
| **HEART** |  |  |
| Have you been diagnosed with high blood pressure? | ✓ |  |
| What was your most recent blood pressure reading? | ✓ |  |
| Have you been diagnosed with high cholesterol? | ✓ |  |
| Have you been diagnosed with low iron? | ✓ |  |
| Have you been diagnosed with Type II diabetes? | ✓ |  |
| Have you been diagnosed with atrial fibrillation? | ✓ |  |
| Are you currently seeing a health professional for this treatment? |  | ✓ |
| Do you eat 2 fruit per day? |  | ✓ |
| Do you eat 5 serves of vegetables per day? |  | ✓ |
| Do you eat lentils and legumes most days in the week? |  | ✓ |
| How often would you eat out per week? |  | ✓ |
| What do you think may stop you from making changes to improve your heart health? |  | ✓ |
| **MIND** |  |  |
| Do you take part in activities that you consider interests or hobbies?  (If yes: how often do you complete these?) |  | ✓ |
| Do you have any hobbies/interests that you would like to explore? |  | ✓ |
| Do you take part in weekly social or community engagements? |  | ✓ |
| Do you have any skills you want to improve? |  | ✓ |
| Are you lonely? |  | ✓ |
| Are you happy with how often to see your friends/family? |  | ✓ |
| Do you have the opportunity to try new things and experiences? |  | ✓ |
| How has COVID impacts you in engaging with others or your community? |  | ✓ |
| How have you remained engaged during restrictions? |  | ✓ |
| What do you think may stop you from making changes to improve your mental health? |  | ✓ |
| **MOOD** |  |  |
| Are you at risk of depression? | ✓ |  |
| Have you ever been diagnosed with depression? |  | ✓ |
| Is a health professional involved in your mental health treatment? |  | ✓ |
| Do you have any support groups to seek assistance? |  | ✓ |
| How has COVID impacts you in engaging with others or your community? |  | ✓ |
| How have you remained engaged during restrictions? |  | ✓ |
| Would you be interested in using therapies to help improve your sleep? |  | ✓ |
| Have you previously taken part in any treatments or programs to improve your mood? |  | ✓ |
| What do you think may stop you from making changes to improve your mood? |  | ✓ |
| **SLEEP** |  |  |
| Have you been diagnosed with a sleep disorder? | ✓ |  |
| Are you suspected of sleep apnoea? | ✓ |  |
| Do you take sleep medication? | ✓ |  |
| Are you at risk of insomnia? | ✓ |  |
| How sleepy are you during the day? | ✓ |  |
| How would you rate your sleep quality? | ✓ |  |
| Work nightshift? | ✓ |  |
| Do you sleep and wake up the same time each day? | ✓ |  |
| Why do you think you have poor sleep? |  | ✓ |
| Can you please describe your sleep?  *Do you sleep and wake at the same time? What about on weekends? What time do you go to bed? Do you snore? Reasons for trouble sleeping? Average hours of sleep per night? Snore?*  *Trouble sleeping?* |  | ✓ |
| May you briefly describe your daily routine 2 hours before going to bed?  *Use of screens before bed. Light on. TV/laptop in bedroom?* |  | ✓ |
| May you please describe your sleeping environment? |  | ✓ |
| What do you think may stop you from making changes to improve your sleep? |  | ✓ |

**Appendix S8:** Weekly check-in questions for intervention participants

**
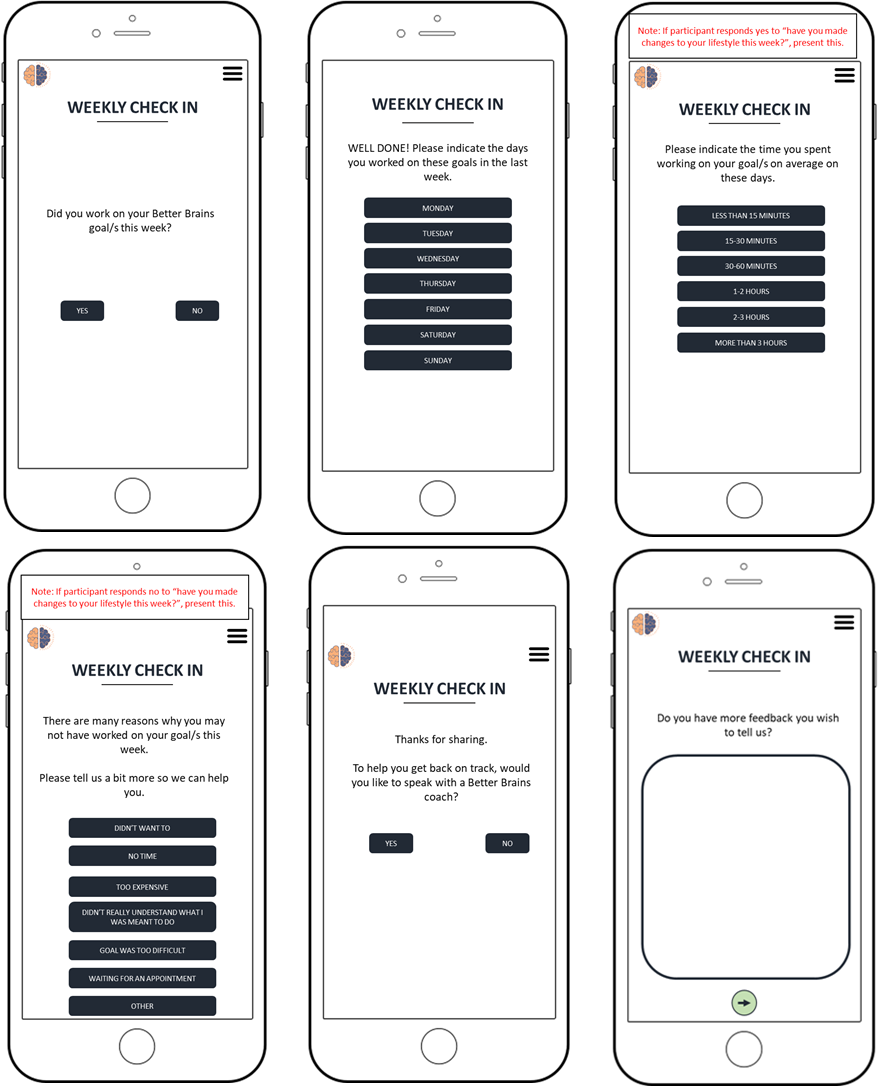
**

**Appendix S9:** Monthly check-in questions for intervention and control participants

The following questions relate to the **past 1 month (28 days)**

**During the past month, on average how many hours per week did you?**

|  | **Definition** | **Never** | **1-2 hours** | **3-4 hours** | **5-6 hours** | **7-8 hours** | **Over 8 hours** |
| --- | --- | --- | --- | --- | --- | --- | --- |
| **Take part in moderate/vigorous physical activity?** | Moderate physical activity: increases your heart rate but you can still talk at the same time (e.g. brisk walking/cycling, dancing, gardening, some housework activities).  Vigorous physical activity increases your heart rate but you cannot talk at the same time (e.g. running, aerobics, swimming, football, basketball, netball). |  |  |  |  |  |  |
| **Engage with cognitively stimulating leisure activities?** | Learning a new skill (e.g. playing an instrument or learning a language). |  |  |  |  |  |  |

**During the past month, please rate**

|  | **Poor** | **Fair** | **Good** | **Excellent** |
| --- | --- | --- | --- | --- |
| **Your sleep quality overall?** |  |  |  |  |
| **How healthy your diet has been?*** |  |  |  |  |

**A healthy diet has been defined as: (using olive oil, eating 5 serves of vegetables (mostly greens) and 2 fruit per day, eating fish at least once weekly, having berries and eating a variety of beans, legumes and grains (e.g. rice, quinoa, buckwheat, etc.) across the week. Wine intake was less than once per week).*

**Mental wellbeing**

**During the past month, how often did you:**

|  | **Always** | **Usually (app. 3 weeks)** | **About half the time (app. 2 weeks)** | **Rarely** | **Never** |
| --- | --- | --- | --- | --- | --- |
| **Feel socially connected with your friends and/or family members?** |  |  |  |  |  |
| **Were you bothered by low mood? (e.g. constant worry, hopelessness, high stress or sadness)?** |  |  |  |  |  |

**Smoking and alcohol**

**During the past month, have there been any changes to your smoking or alcohol consumption?**

|  | **Increased** | **Decreased** | **No change** |
| --- | --- | --- | --- |
| **Alcohol** |  |  |  |
| **Smoking** |  |  |  |

**Access to health services**

**During the past month, please indicate if you have seen any of the following health professionals (please tick all that apply):**

|  | **Yes** |
| --- | --- |
| **GP** |  |
| **Physiotherapist** |  |
| **Psychologist** |  |
| **Dietitian/nutritionist** |  |
| **Exercise physiologist** |  |
| **Other, please specify below** |  |
| **No health professionals seen** |  |

**COVID-19**

|  | **Not at all** | **Somewhat** | **A lot** |
| --- | --- | --- | --- |
| **To what extent have COVID-19 restrictions impacted your responses above?** |  |  |  |

**Appendix S10:** Referral letters according to participant presentation and module in the BetterBrains program

| **Domain** | **Participant presentation** |
| --- | --- |
| BetterHearts | Referral for a Heart Health assessment |
|  | Referral for alcohol use review |
|  | Referral for smoking cessation plan |
|  | Strategies focus on medical management |
|  | Referral for smoking cessation plan + alcohol use review |
|  | Request referral for an allied health service through a GPMP |
|  | Direct referral to a private provider |
| BetterMood | Referral to GP for risk factor review |
|  | Referral to GP for MH review and creation of Mental Health Care Plan |
|  | Referral to GP for psychiatry referral |
|  | Pharmacotherapy/Medication Adherence |
|  | Direct referral to a private provider |
| BetterSleep (no sleep disorder) | Obstructive sleep apnoea + advanced sleep phase |
|  | Obstructive sleep apnoea + insomnia |
|  | Obstructive sleep apnoea + sleep medical |
|  | Advanced sleep phase + insomnia |
|  | Advanced sleep phase + sleep medication |
|  | Sleep medication + insomnia |
| BetterSleep (sleep disorder diagnosis confirmed) | Risk of sleep apnoea. |
|  | Risk of both sleep apnoea + sleep medication taking. |
|  | Risk of both sleep apnoea + insomnia. |
|  | Risk of insomnia. |
|  | Risk of both insomnia + sleep medication taking. |
|  | Risk of sleep medication taking. |
|  | At risk of all insomnia + sleep apnoea + taking sleep medication. |
|  | Advanced sleep phase protocol completed and is ineffective. |
|  | Insomnia treatment completed and is ineffective. |
|  | Medication, medical condition or eye condition sensitive to light |
|  | Risk of obstructive sleep apnoea |
|  | Risk of obstructive sleep apnoea + taking sleep medications. |
|  | At risk of both obstructive sleep apnoea and insomnia. |
|  | At risk of insomnia. |
|  | At risk of insomnia + taking sleep medication. |
|  | Taking sleep medication. |
|  | At risk of sleep apnoea and insomnia + taking sleep medication. |

**Appendix S11:** Managing psychological risk


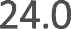

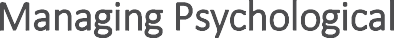

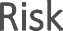


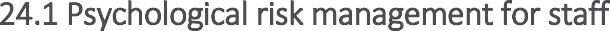


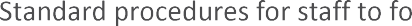

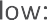


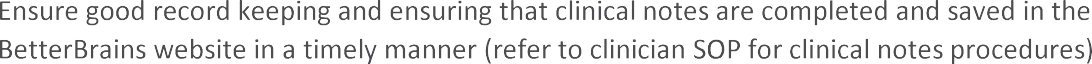


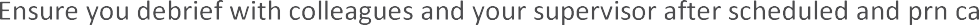

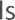

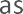

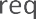

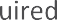

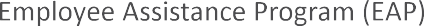

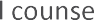

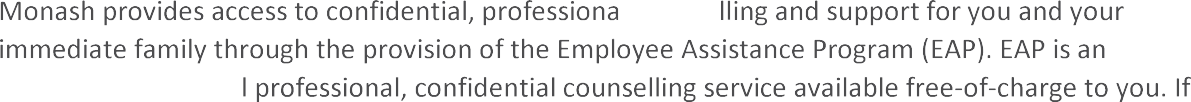

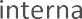

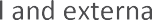


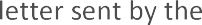


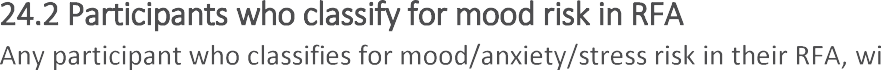

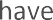

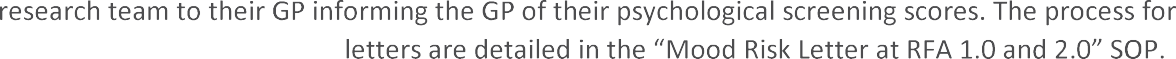

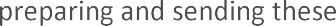


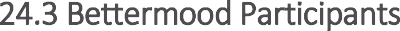


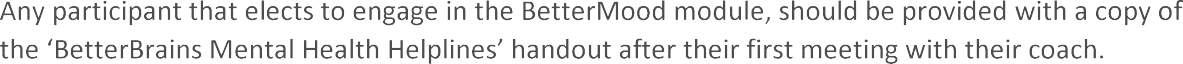


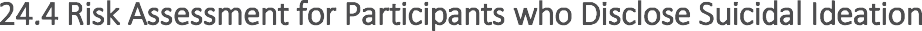


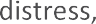

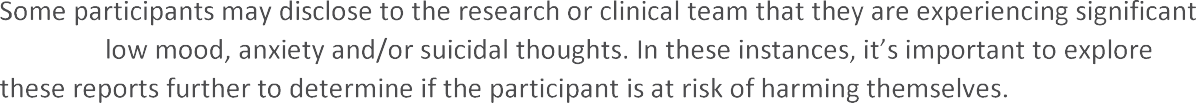

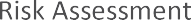

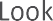

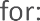


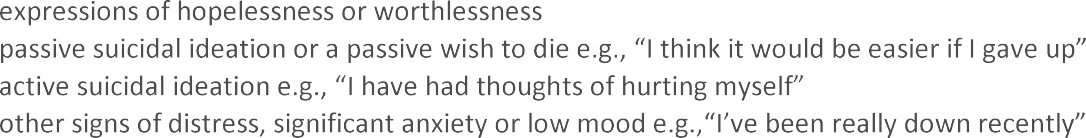


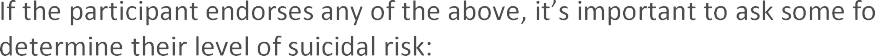

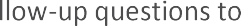


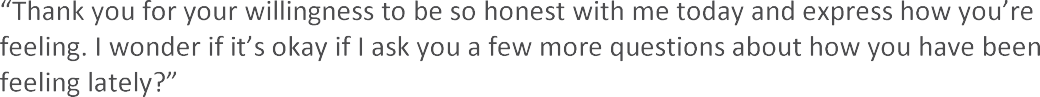


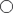

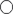

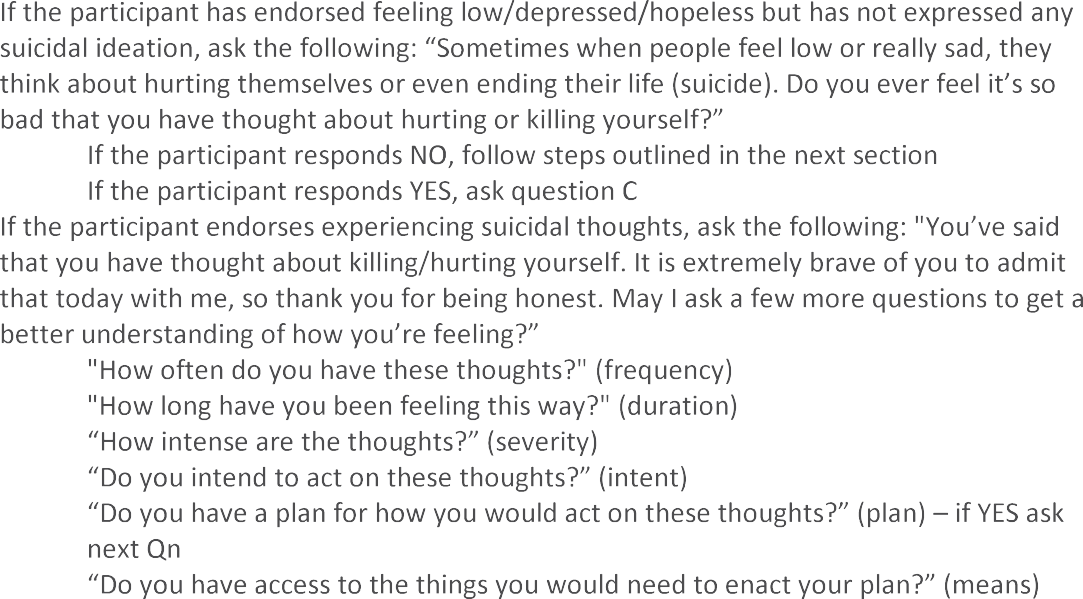

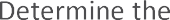

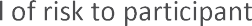


| 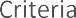 | 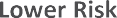 | 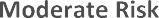 | 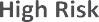 |
| --- | --- | --- | --- |
| 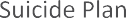 | 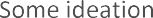  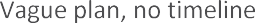 | 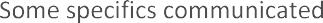 | 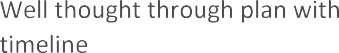 |
| 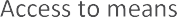 | 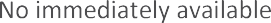 | 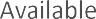 | 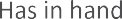 |
| 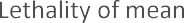 | 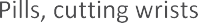 | 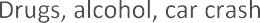 | 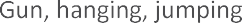 |
| 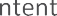 | 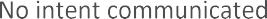 | 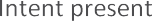 | 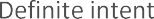 |
| 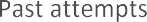 | 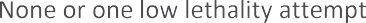 | 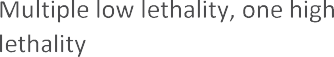  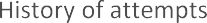 | 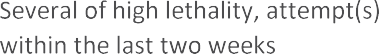 |
| 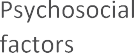 | 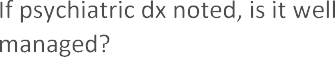  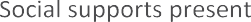  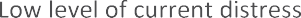  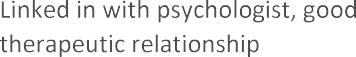 | 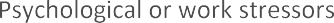  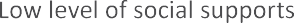  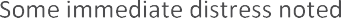  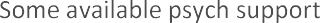 | 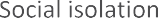  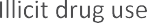  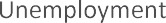  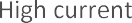 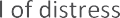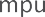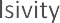 |


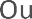

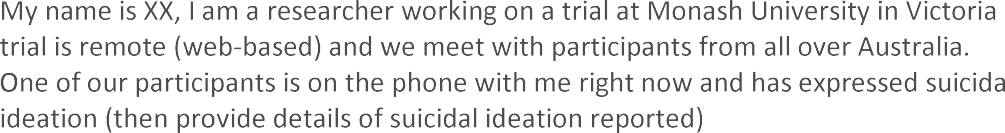

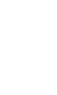

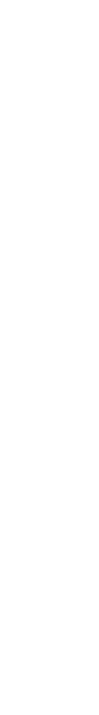

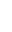

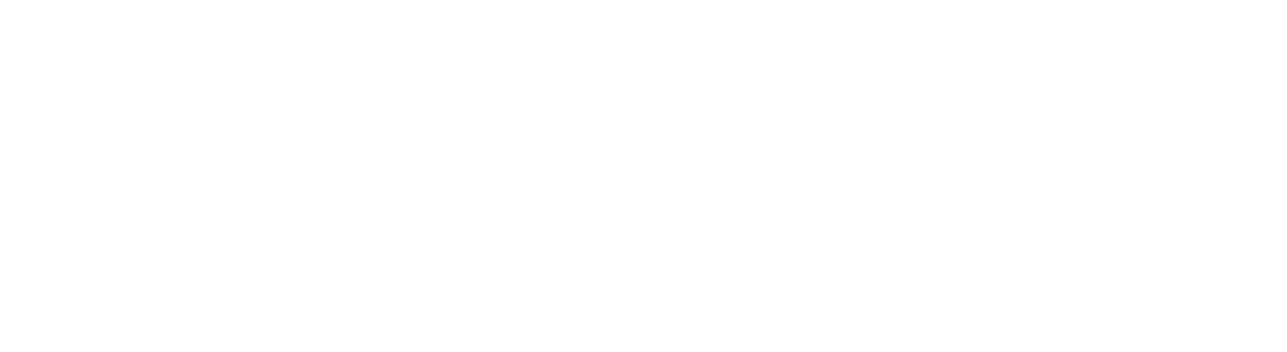

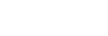

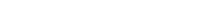

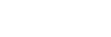

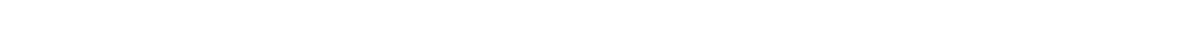

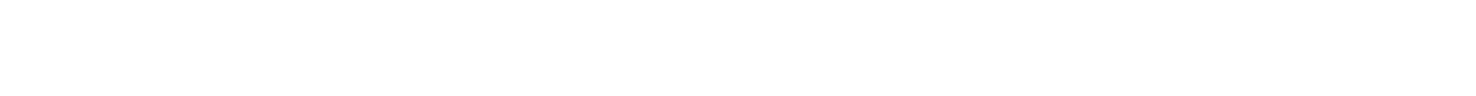

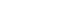

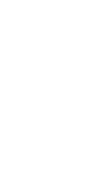

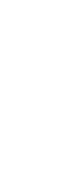

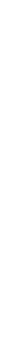


**Appendix S12:** BetterBrains coaches post- training assessment form

Thank you for participating in the BetterBrains clinician training. The following are some questions we would like you to answer as a way of measuring the application of the training content and usability of the Clinician SOPs.

**BetterBrains overall**

1. Briefly describe the overall aim of the BetterBrains trial:

*To test the hypothesis that a behaviour modification intervention program prevents cognitive decline in community-dwelling, middle-age adults with a family history of dementia.*

1. Which are the 4 BetterBrains modules:

*BetterMood, BetterSleep, BetterMind, BetterHearts*

1. Which is true?

- *Recommended goals and strategies need to be person-centred but also dependent on the risk factors management pathway, which can be determined using the 4 BetterBrains modules and clinician SOPs.*
- Recommended goals and strategies are person-centred only. Using a risk factor management pathway does not apply.
- Recommended goals and strategies are only dependent on the risk factor management pathway and are not person-centred.

1. Within what time frame should a GP letter be sent following a call?

*Must be sent on the same day as the call.*

1. What should the clinician do when a participant disengages with BetterBrains?

- *Record it on the participant disengaged excel spreadsheet*
- Email the RA team
- Do nothing

1. What should you do at the end of a call according to motivational interviewing principles:

- Thank them for their time
- *Summarise the main points that you discussed and their action plan (next steps). Also, ask the participant if you have missed anything and provide a chance to answer any of their questions.*
- Agree that change is hard

1. How many calls does a participant need to complete over the Better Brains program?

- 4
- *6*
- 8
- 10

1. How long does the BetterBrains program run for?

- 6 months
- *12 months*
- 24 months

1. Which program is used to help book in participant calls?

*Calendly*

1. How do clinicians and participants communicate?

- Email
- *Internal BetterBrains messaging system*
- Mobile

**Baseline call**

1. How do you know a participant is ready for the clinician to contact them to schedule the baseline call?

*After the participant has completed baseline, and they have been assigned to your patient list (clinician will see participant on their client list).*

1. Please fill in the blank: Clinicians should aim to contact their participants within __________ of randomisation/alert to welcome message within:

- The same day of the alert
- 48 hours
- *1 week*

1. Name four things that must be completed during a baseline call:

*1. Introduce yourself*

*2. Answer any BB-related questions*

*3. Complete initial assessment*

*4. discuss personalised risk-factors to cognitive decline*

1. Name four things that are completed during a FUP call:

*1. review current goals and strategies*

*2. identify barriers and use MI to work with the participant and overcome these barriers*

*3. Edit old or develop new goals as required*

*4. Answer any questions the participant may have*

**Strategy recommendations and goal development**

1. What is a SMART goal? Explain the acronym and provide an example of a SMART goal.

*S = specific, M = measurable, A = achievable, R = relevant, T = timely*

*An example of a SMART goal: To improve physical activity level, aim to complete 6000 steps per day (walking) over the next 4 weeks.*

1. Which module needs to be the most attentive to the risk factor management pathway?

- Better Heart
- BetterMind
- BetterMood
- *BetterSleep*

1. What should you check before focusing on the BetterSleep module, if the participant wishes to focus on sleep?

- *If they have an official sleep disorder, if so, if they are receiving any sleep treatment and how long ago did they receive any sleep treatment or see a sleep specialist?*
- Has a high BMI
- Had high blood pressure
- Nothing to check

1. If a participant qualifies for the advanced sleep phase and wants to focus on improving the timing of their sleep, what is the one thing you MUST check for:

- Their weight
- Mood
- *Obstructive sleep apnoea risk*
- Hypertension

**Appendix S13:**BetterBrains coach training content

| Day 1 | - Introduction to BetterBrains - Coach roles and responsibilities - Module overview - Participant journey overview - RCT design and basic research considerations |
| --- | --- |
| Day 2 | - Data collection and outcome data overview - Participant privacy and confidentiality agreement - Scheduling and completing an online coaching session - Risk factor management pathways - Adverse and serious adverse event standard operating procedures - Referral letters |
| Day 3 | - BetterBrains program evaluation overview - Coach fidelity measures - Coach safety and wellbeing - Motivational interviewing training |
| Case-study day | - Case studies run through in pairs (1 x initial and 1 x follow up session each) - Informal feedback provided by the senior coach |
| Observation day | - Observing the senior coach during an online initial and follow-up session - Question and answer session |
| Coach performance | - Senior coach to complete observations every 2 months - Weekly export of coach performance and participant engagement - Feedback during weekly meetings |

**Appendix S14:**BetterBrains coach competencies to ensure study fidelity (executed as intended)

| **Competency** | **Time of completion** | **Content to be completed** | **Purpose** |
| --- | --- | --- | --- |
| Post-training competency | Within 1 week of when the 3-day training is completed | 1. Competency assessment (Appendix S11) - assessed by the senior coach  - higher score indicates greater competency understanding. - score <50% requires repeated assessment and competency checklist completed within 1 week. - Sessions are completed online or in-person  1. Post-training checklist (Appendix S14) - monitor knowledge of the BetterBrains program and motivational interviewing. 2. Post-training evaluation form (Appendix S15) | Assess coach's understanding of the main BetterBrains components and considerations for person-centred care |
| Ongoing fidelity checking | Within 7 business days of shadowing the senior coach and every 2 months following. | 1. Coaching fidelity checklist (Appendix S16):  - assesses implementation of motivational interviewing principles and core tasks during the initial and follow-up session - assessed by the senior coach. - higher score indicates greater protocol fidelity. - feedback provided by the Senior BetterBrains coach. | To ensure coaches are consistently implementing BetterBrains according to protocol across the 12-month timeline |

**Appendix S15:**Post-training BetterBrains competency checklist

**Low = 1, adequate = 2, High = 3**

| **Data collector Name:** | | |
| --- | --- | --- |
| **Task list** | **Competent level** | **Comment** |
| **Practical tasks to be completed post coach training Date of competency:** | | |
|  | **Score** |  |
| 1. Have access to a Monash email account and is able to send a test email to the senior coach. |  |  |
| 1. Received login details and able to enter the BB coach webpage. |  |  |
| 1. Able to briefly explain how to access reminders (welcome, baseline and FUP calls) |  |  |
| 1. Able to complete a baseline eCRM |  |  |
| 1. Able to complete a FUP eCRM |  |  |
| 1. Able to access the GP letters tab and briefly explain why this is important. |  |  |
| 1. Able to access own coach profile page and alter availability times. |  |  |
| 1. Able to access own Calendly account and can send through each relevant BetterBrains call link to a participant. |  |  |
| 1. Able to access own participant list and know which calls are booked in for the day |  |  |
| 1. Able to access own email calendar and link this to Calendly |  |  |
| 1. Able to show that have the BB coach protocol in easy access |  |  |
| **Knowledge of BB intervention post coach training Date of competency:** | | |
|  | **Score** |  |
| 1. Able to explain the aims of the BB trial |  |  |
| 1. Able to identify and briefly describe the 4 BB modules |  |  |
| 1. Able to identify the risk factors that correspond to each module |  |  |
| 1. Able to provide an example of a SMART goal |  |  |
| 1. Able to briefly explain the protocol for an Adverse Event or Serious Adverse Event (for the coach) |  |  |
| 1. Able to briefly explain when a GP letter should be sent and how |  |  |
| 1. Able to access the internal messaging system and briefly explain when coaches will need to use this when contacting participants. |  |  |
| 1. Able to explain the coach protocol when a participant disengages or re-engages |  |  |
| 1. Able to describe the 4 reasons that turn a PRN call into a FUP call |  |  |
| 1. Able to explain the time period of when a participant should be contact both post randomisation and when an alert pops up |  |  |
| **Task list** | **Competent level** | **Comment** |
| **Knowledge of MI in BB post coach training** | | |
|  | **Score** |  |
| 1. The 4 stages of MI |  |  |
| 1. The 5 stages of change |  |  |
| 1. What constitutes a reflection (simple, complex, double-sided) |  |  |
| 1. What constitutes an open-ended question |  |  |
| 1. What word shows ambivalence and how to react to this |  |  |
| 1. What to do when wanting to provide advice |  |  |
| 1. How to build an evoking question |  |  |
| 1. How to start change talk |  |  |
| 1. The main action points when starting a call |  |  |
| 1. The main action points when summarising a call |  |  |

**‘**

**Appendix S16:** BetterBrains coaches post- training evaluation form

Thank you for participating in the BetterBrains clinician training. The following are some questions to help us evaluate the BetterBrains clinician training program.

1. How confident do you feel starting as a BetterBrains clinician following the training?

| 1 | 2 | 3 | 4 | 5 | 6 | 7 | 8 | 9 | 10 |
| --- | --- | --- | --- | --- | --- | --- | --- | --- | --- |
| *Not confident at all* |  |  |  |  |  |  |  |  | *Extremely confident* |

Why is this?

1. Please list some strengths of the training session
2. Please list the areas/points that may need improving
3. Please state any questions or gaps you think should have been included in the training
4. How supported do you feel following the BetterBrains clinician training?

| 1 | 2 | 3 | 4 | 5 | 6 | 7 | 8 | 9 | 10 |
| --- | --- | --- | --- | --- | --- | --- | --- | --- | --- |
| *Not supported at all* |  |  |  |  |  |  |  |  | *Extremely supported* |

1. How engaging do you feel was the BetterBrains clinician training?

| 1 | 2 | 3 | 4 | 5 | 6 | 7 | 8 | 9 | 10 |
| --- | --- | --- | --- | --- | --- | --- | --- | --- | --- |
| *Not supported at all* |  |  |  |  |  |  |  |  | *Extremely supported* |

Thank you! 😊

**Appendix S17:**Coaching fidelity checklist (completed every 2 months)

**Not applicable = 0, Low = 1, adequate = 2, High = 3**

Coach:

| **Task list** | **Competent level** | **Comment** |
| --- | --- | --- |

| **Tasks to be observed during call Date of competency:** | | |
| --- | --- | --- |
|  | **Score** |  |
| 1. Coach looks over participant record before call |  |  |
| 1. Coach starts the timer |  |  |
| 1. Coach introduces themselves as a BB coach (baseline only) |  |  |
| 1. Coach explain a minimal of 6 calls must be completed across the 12-month intervention (baseline only) |  |  |
| 1. Coach explains communication methods during BB (baseline only) |  |  |
| 1. Coach asks the participant if they have any questions |  |  |
| 1. Coach clearly outlines structure of session |  |  |
| 1. Coach clearly describes the person’s risk factors (baseline only) |  |  |
| 1. Coach asks open-ended questions |  |  |
| 1. Coach completes relevant clinical assessment, if required |  |  |
| 1. Coach clearly explains their view of the risk factors and how it is linked to a condition or cognitive decline. (RPAD-1) |  |  |
| 1. Goal and strategy development is person-centred |  |  |
| 1. Coach queries barriers to goal attainment and works with the person to help overcome these. (RPAD-2) |  |  |
| 1. Coach queries facilitators to goal attainment and works with the person to best making these facilitators possible. (RPAD-4) |  |  |
| 1. Coach enters in COVID-19 questions (if required) |  |  |
| 1. Coach refers to GP or community services (if required) |  |  |
| 1. Coach provides information sheets (if required) |  |  |
| 1. Next BB call is confirmed or Calendly invite sent |  |  |
| 1. Coach thanks person for their time to end call |  |  |
| 1. Coach stops timer and exports call time |  |  |
| 1. Call time within 45 or 30 min allocation |  |  |
| 1. Coach enters in all required information into the eCRM with 100% accuracy |  |  |
| 1. Coach ‘additional notes’ are appropriate, with 100% accuracy |  |  |
| 1. Coach adds in note taking admin time within the eCRM |  |  |
| 1. Coach submits the eCRM |  |  |
| 1. Coach uses lay language or matches the patient’s level of technical language |  |  |
| **Motivational interviewing** |  |  |
| 1. Coach shows a marked and consistent effort to increase the depth, strength, or momentum of the person’s language in favour of change (Cultivating change talk) |  |  |
| 1. Coach shows a marked and consistent effort to decrease the depth, strength, or momentum of the person’s language in favour of difficulty or undesirability of change. (Softening sustain talk) |  |  |
| 1. Coach actively fosters and encourages power sharing in the interaction in such a way that the person’s contributions substantially influence the nature of the session (Partnership) |  |  |
| 1. Coach shows evidence of deep understanding of client’s point of view, not just for what has been explicitly stated but what the person means and has not yet said. (Empathy) |  |  |
| 1. The coach gives information, educates, provides feedback or expresses a professional opinion without persuading, advising or warning and keeps the tone of the conversation neutral. The coach does not imply that the person must act on it. (Giving information) (RPAD-2) |  |  |
| 1. The coach asks the person for permission to provide advice, opinion, tips, ideas, before doing so (Permission). |  |  |
| 1. The coach makes overt attempts to change the person’s opinions, attitudes, or behaviour using tools such as logic, compelling arguments, self-disclosure, or facts (and the explicit linking of these tools with an overt message to change). Information, ideas, opinions, tips may be biased. |  |  |
| 1. The coach asks open-ended questions and minimal closed-ended questions (RPAD-8) |  |  |
| 1. The coach uses simple reflections to convey understanding or facilitate person-coach exchange. (RPAD-9) |  |  |
| 1. The coach uses complex reflections to convey deeper or more of a complex picture to what the person may have stated. (RPAD-9) |  |  |
| 1. The coach uses double-sided reflections to convey deeper or more of a complex picture to what the person may have stated as well as try to stimulate change talk. (RPAD-9) |  |  |
| 1. The coach positively points out the person’s strength, efforts, intentions or worth and does not over emphasis the same affirmations across the conversation. (Affirmation) |  |  |
| 1. The coach attempts to share power or knowledge with the person by understanding the person’s current state of knowledge (Seeking collaboration) |  |  |
| 1. The coach clearly focuses the responsibility toward the person to make decisions about and actions pertaining to change. The coach highlights the person’s sense of control, freedom of choice, personal autonomy, or ability or obligation to decide about their attitudes and actions. *These are not statements that specifically emphasize the client’s sense of self-efficacy, confidence, or ability to perform a specific action.* |  |  |
| 1. The coach actively asks the person to agree and obtain commitment to change according to the person-centred goals/strategies developed. (RPAD-3) |  |  |
| 1. The coach summarises the conversation at the end of the conversation and briefly recaps the next steps. |  |  |
| 1. The coach gives the person the opportunity to ask questions by asking ‘Any questions?’ If so, the coach engages in discussion about the question. (RPAD- 7) |  |  |
| 1. The coach asks whether there is anything that they have missed or want to clarify. The coach ensures the person understand the goal/strategies to work towards. (RPAD-5) |  |  |
| 1. Coach is sending welcome messages on the day of alert. |  |  |
| 1. Coach is sending Calendly and zoom invites to participants as needed. |  |  |
| 1. Coach is contacting the participant within 48 hours of the booking alert. |  |  |
| 1. Coach is completing notes with 100% accuracy and the required detail |  |  |
| 1. Coach is referring to GPs when required |  |  |
| 1. Coach is completing GP letters on the same day of a completed call using the BB templates. |  |  |
| 1. Coach is calling participants at the scheduled call time |  |  |
| 1. Coach communicating to AE/SAE to the RA team when needed |  |  |
| 1. Coach is maintaining an adequate participant load |  |  |
